# Comparative Assessment of Salivary Neutrophil Gelatinase-Associated Lipocalin Levels (NGAL) Among Subjects With Periodontitis, Liver Cirrhosis, and Healthy Individuals: A Cross-Sectional Study

**DOI:** 10.7759/cureus.110562

**Published:** 2026-06-09

**Authors:** Sayali S Dongare, Sameer A Zope, Girish Suragimath, Siddhartha Varma, Apurva V Kale

**Affiliations:** 1 Department of Periodontology, School of Dental Sciences, Krishna Vishwa Vidyapeeth (Deemed to be University), Karad, IND

**Keywords:** biomarker, liver cirrhosis, neutrophil gelatinase-associated lipocalin, oral–systemic link, periodontitis, saliva

## Abstract

Background: Periodontitis and liver cirrhosis are chronic inflammatory disorders characterized by dysregulated immune responses and an increased systemic inflammatory load. Neutrophil gelatinase-associated lipocalin (NGAL) is a neutrophil-derived biomarker implicated in inflammation, tissue injury, and systemic disease. The relationship between salivary NGAL and periodontal health has not been previously studied in individuals with liver cirrhosis.

Aim: To evaluate and compare salivary NGAL levels among patients with liver cirrhosis with and without periodontitis, periodontitis alone, and healthy controls.

Materials and methods: This cross-sectional study included 40 participants divided into four groups (n=10 each): liver cirrhosis with periodontitis, liver cirrhosis only, periodontitis only, and healthy controls. Periodontal status was evaluated using standardized clinical parameters in accordance with the 2017 American Academy of Periodontology (AAP) classification criteria. Unstimulated whole saliva samples were obtained, and salivary NGAL concentrations were quantified using enzyme-linked immunosorbent assay (ELISA). Intergroup comparisons were analyzed using one-way analysis of variance (ANOVA).

Results: Salivary NGAL levels differed significantly among the study groups (p<0.001). The highest NGAL levels were observed in patients with liver cirrhosis and periodontitis, followed by liver cirrhosis alone, periodontitis alone, and healthy controls. These findings indicate an additive inflammatory effect when periodontal disease coexists with liver cirrhosis.

Conclusion: Salivary neutrophil gelatinase-associated lipocalin (NGAL) levels were significantly increased in patients presenting with both liver cirrhosis and periodontitis, indicating an enhanced cumulative systemic and periodontal inflammatory burden. Salivary NGAL may serve as a potential non-invasive biomarker linking periodontal inflammation and liver disease. Further longitudinal investigations involving larger sample populations are required to substantiate its clinical applicability and diagnostic utility.

## Introduction

Periodontitis is a chronic, multifactorial inflammatory disease affecting the tooth-supporting tissues, characterized by progressive destruction of the periodontal ligament and alveolar bone, which, if left untreated, can ultimately result in tooth loss. It is initiated by dysbiotic microbial biofilms and perpetuated by a disproportionate host inflammatory response. Epidemiological data indicate that severe periodontitis affects approximately 11.2% of the global population, making it one of the most prevalent chronic inflammatory conditions worldwide and a significant public health concern [1,2]. As inflammation advances, the epithelium lining deep periodontal pockets becomes ulcerated and permeable, providing a gateway for periodontal pathogens, their virulence factors, and inflammatory mediators to disseminate into the bloodstream [3]. This systemic translocation establishes a biological link between periodontitis and distant organ systems, contributing to systemic inflammatory burden. Chronic liver disease (CLD) represents a progressive, long-standing deterioration of liver structure and function due to diverse etiologies, including alcohol abuse, viral hepatitis, metabolic disorders, toxin exposure, and autoimmune diseases [4]. As liver injury progresses, chronic inflammation and fibrosis lead to architectural distortion, culminating in cirrhosis, the end stage of chronic liver disease. Liver cirrhosis (LC) is a major global health problem and currently ranks as the 11th leading cause of death worldwide, reflecting its clinical and socioeconomic impact [5]. Chronic liver disease and cirrhosis are associated with systemic inflammation, immune dysregulation, oxidative stress, and altered gut-liver axis interactions. An emerging body of evidence indicates a bidirectional relationship between chronic periodontal inflammation and liver disorders, particularly non-alcoholic fatty liver disease, steatosis, and cirrhosis. Oral microorganisms such as *Porphyromonas gingivalis* and *Aggregatibacter actinomycetemcomitans*, along with lipopolysaccharides (LPS) and pro-inflammatory cytokines released from periodontal tissues, can reach the liver via the systemic circulation or enteric routes, causing hepatic oxidative stress, lipid peroxidation, and metabolic disturbances [6]. This mechanistic pathway may contribute to liver steatosis and perpetuate hepatic inflammation. Studies further demonstrate that individuals with periodontitis exhibit changes in gut microbiota, leading to intestinal dysbiosis. Conversely, cirrhosis is associated with increased oral-gut microbial translocation, suggesting a disrupted oral-gut-liver axis [7]. For instance, elevated levels of *Fusobacteria*, commonly associated with periodontal disease, have been reported in the saliva of cirrhotic patients, while another study observed that cirrhosis patients typically present with multiple teeth exhibiting deep periodontal pockets (≥6 mm), indicating a higher prevalence of periodontitis [8]. Neutrophil gelatinase-associated lipocalin (NGAL), also known as lipocalin-2, is a 25-kDa low-molecular-weight glycoprotein expressed in neutrophils, renal tubular cells, hepatocytes, epithelial cells of the gastrointestinal tract, and various immune cells. It plays a significant role in innate immunity through iron sequestration, bacteriostatic activity, and modulation of oxidative stress responses [9]. NGAL is markedly upregulated in conditions involving infection, inflammation, and tissue injury. Clinically, NGAL is recognized as a sensitive and early biomarker for acute kidney injury and has demonstrated diagnostic utility in distinguishing acute tubular necrosis from other forms of renal injury, particularly in patients with liver cirrhosis. Furthermore, increased NGAL expression has been observed in hepatic injury and has been implicated in the pathogenesis of hepatic inflammation, fibrosis, and cirrhosis [10]. Although NGAL has been extensively studied in serum and urine, its expression in saliva is gaining attention due to the non-invasive nature of saliva collection and its potential to reflect both local and systemic inflammatory states. Given that NGAL is produced by neutrophils and significantly increases during bacterial infection or inflammatory activation, salivary concentrations may serve as indicators of periodontal inflammation as well as systemic disease burden [11]. Despite the biological plausibility linking periodontitis, liver cirrhosis, and NGAL, no previous studies have evaluated salivary NGAL levels in patients with liver cirrhosis in relation to periodontal status. The present study was based on the hypothesis that salivary NGAL levels would be elevated in individuals with liver cirrhosis, periodontitis, or both conditions compared with healthy controls, reflecting an increased inflammatory burden. Therefore, this study aimed to evaluate and compare salivary NGAL levels among patients with liver cirrhosis with and without periodontitis, periodontitis alone, and healthy controls. By exploring the relationship between these conditions and salivary NGAL expression, this study seeks to enhance understanding of the oral-systemic interplay in liver disease and assess the potential of NGAL as a non-invasive biomarker of disease-associated inflammation.

## Materials and methods

The present cross-sectional study was undertaken in the Department of Periodontology, School of Dental Sciences (KVV), Krishna Vishwa Vidyapeeth, Karad. Approval of the study protocol was obtained from the Institutional Ethics Committee. The study was conducted over a period of six months, from November 2024 to April 2025.

Sample size selection

**Figure 1:**
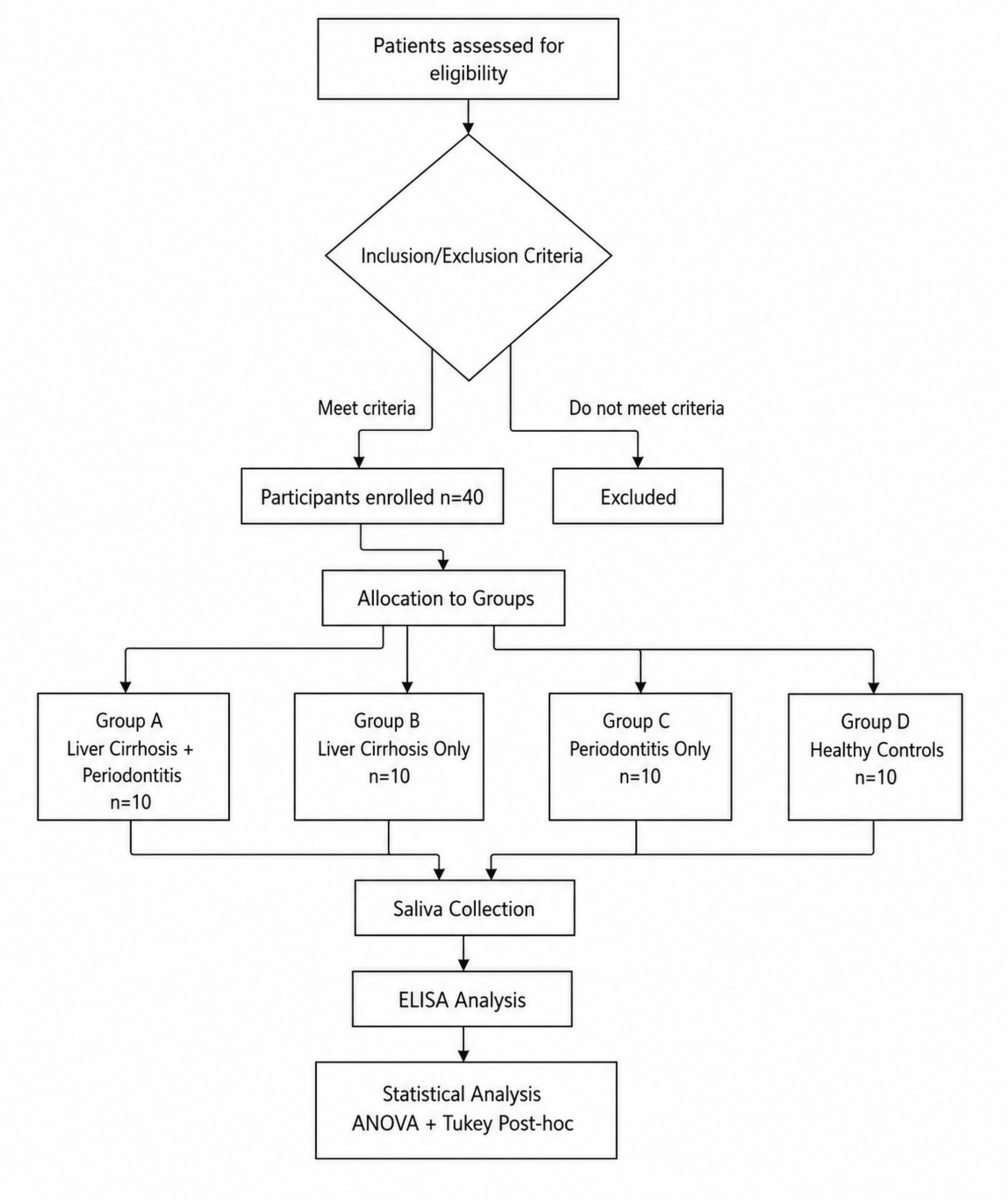
Participant recruitment and allocation flow diagram

A total of 40 patients (n=40) who reported to the Department of Periodontology and the Department of General Medicine were randomly included in the study (Figure 1). The sample size of 40 patients (n=10 in each of the four groups) was derived using the formula given below. The sample size was calculated using the formula: \[ N = \frac{(Z_{1-\alpha/2} + Z_{\beta})^{2}(SD_1 + SD_2)^{2}}{(\mu_1 - \mu_2)^{2}} \] 

Inclusion criteria

Participants aged 20-60 years, both male and female, were included and divided into four groups. Group A (n=10) included patients diagnosed with liver cirrhosis and have periodontitis. Group B (n=10) included patients diagnosed with liver cirrhosis. Group C (n=10) included patients with stage II/III and IV periodontitis, and Group D (n=10) included healthy subjects (Figure 1). 

Exclusion criteria

Patients who had used any medications known to influence periodontal tissues and those with a history of periodontal therapy within the previous three months were excluded from the study. Additionally, any patient who did not provide consent to participate was not enrolled in the study.

Clinical examination

Clinical evaluation was conducted in the Departments of Periodontology and General Medicine. Periodontal status was assessed using standard clinical parameters, including PPD, CAL, BOP, and plaque index, and subjects were classified with or without periodontitis according to the 2017 AAP Classification. Periodontitis subjects having stage II to IV were included in the study. Liver cirrhosis patients were assessed and diagnosed in the Department of General Medicine using clinical examination, liver function tests, ultrasonography, and documented medical history, following the Child-Pugh classification criteria. Only medically stable cirrhotic patients meeting the study criteria were included.

Sample collection

Unstimulated whole saliva was collected from each participant using the passive drool method. An unstimulated 2 mL sample was obtained between 10:00 AM and 12:00 PM following a modification of the Navazesh method. Participants were instructed to avoid eating, drinking, smoking, or performing oral hygiene procedures for one hour prior. To prevent blood contamination, clinical parameters were recorded at least one hour before collection. Each participant swallowed once and then allowed saliva to drain passively for 5 minutes into sterile, pre-labeled microcentrifuge tubes. Samples from liver cirrhosis patients were collected with additional care to avoid discomfort.

Sample storage

Collected samples were centrifuged for 15 minutes at 1,000 rpm at 2-8°C. All the particulates were removed and stored. Collected saliva was immediately stored in an aliquot at <-80°C. Samples were analyzed within two months of collection.

Biomarker analysis

**Figure 2:**
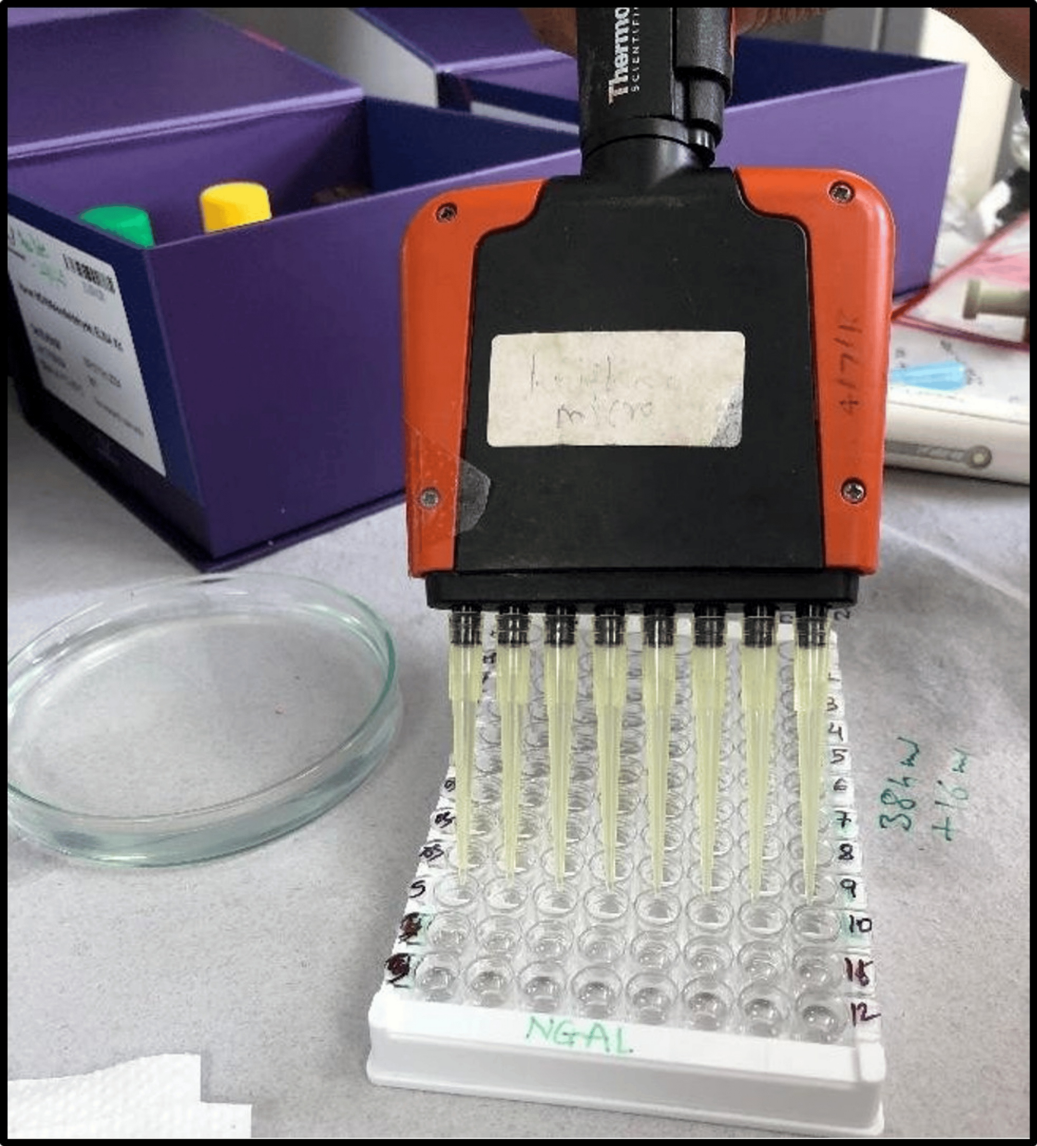
Addition of NGAL reagents into salivary supernatant samples before ELISA analysis

The concentration of salivary neutrophil gelatinase-associated lipocalin (NGAL) was determined using enzyme-linked immunosorbent assay kits (EasyStep Human NGAL ELISA Kit, ELK Biotechnology, USA). The analysis was conducted at the Microbiology Laboratory, Krishna Vishwa Vidyapeeth, a deemed-to-be university in Karad (Figure 2).

Statistical analysis

The results of biomarker analysis were subjected to statistical analysis using IBM Corp. Released 2015. IBM SPSS Statistics for Windows, Version 21. Armonk, NY: IBM Corp. Comparison of various parameters between the groups was done using the analysis of variance formula test (ANOVA).

## Results

A total of 40 participants were included in the study and were divided into four groups of ten each. Comparison of mean age among the groups showed that Group A had the highest mean age at 65.5 years, followed by Group B with a mean age of 55.5 years. Group C demonstrated a mean age of 51.9 years, while Group D showed the lowest mean age at 43.5 years. Statistical analysis using one-way ANOVA revealed a significant difference in mean age between the groups (F = 18.42, p = 0.0003**).

Table 1 indicates that the comparison of mean age among the four study groups revealed a statistically significant difference. Group A had the highest mean age (65.5 ± 6.80 years), followed by Group B with a mean age of 55.5 ± 7.10 years and Group C with a mean age of 51.9 ± 8.20 years. Group D showed the lowest mean age (43.5 ± 7.50 years). One-way ANOVA demonstrated a highly significant difference in mean age across the groups (F = 18.42, p = 0.0003), indicating that age distribution varied significantly among the study populations.

**Table 1:** Comparison of mean age between various groups One-way ANOVA F = 18.42, p = 0.0003**

Groups	Mean	SD	SE	Minimum	Maximum
Group A – Liver Cirrhosis patients who have Periodontitis (n=10)	65.5	6.80	2.15	55.2	75.8
Group B – Liver Cirrhosis Only (n=10)	55.5	7.10	2.25	44.9	66.1
Group C – Periodontitis (n=10)	51.9	8.20	2.59	38.5	65.3
Group D – Healthy (n=10)	43.5	7.50	2.37	31.8	55.2

Table 2 illustrates the comparison of gender distribution among the four study groups, which showed a statistically significant difference. The LC with periodontitis group comprised 10 (100%) males. Similarly, the liver cirrhosis only group predominantly consisted of males (9 [90%]), with a small proportion of females (1 [10%]). In the periodontitis group, males accounted for 6 (60%) participants, while females accounted for 4 (40%). The healthy control group demonstrated an equal gender distribution, with 5 (50%) males and 5 (50%) females. The Chi-square test revealed a significant variation in gender distribution across the groups (χ² = 12.14, p = 0.0069), indicating that gender distribution differed significantly among the study populations.

**Table 2:** Comparison of gender distribution between various groups Chi-square test = 12.14, p = 0.0069

Groups	Male n (%)	Female n (%)	Total n (%)
Group A -Liver Cirrhosis patients who have Periodontitis	10 (100.0%)	0 (0.0%)	10 (100)
Group B- Liver Cirrhosis Only	9 (90.0%)	1 (10.0%)	10 (100)
Group C- Periodontitis	6 (60.0%)	4 (40.0%)	10 (100)
Group D- Healthy	5 (50.0%)	5 (50.0%)	10 (100)

Table 3 demonstrates significant differences in periodontal clinical parameters among the study groups (p < 0.001). Groups A (liver cirrhosis with periodontitis) and C (periodontitis only) exhibited the highest OHI-S scores, Russell's Periodontal Index scores, probing pocket depths (PPD), and clinical attachment loss (CAL), indicating moderate-to-severe periodontal destruction. In contrast, Group B (liver cirrhosis only) showed poor oral hygiene but minimal periodontal destruction, while healthy controls (Group D) exhibited the lowest periodontal scores. These findings confirm that periodontal disease was primarily confined to Groups A and C and validate the clinical grouping of study participants.

**Table 3:** Intergroup comparison among all periodontal parameters using ANOVA test *One-way ANOVA; statistically significant at p < 0.05. PPD: probing pocket depths, CAL: clinical attachment loss

Parameter	Group A (LC + Periodontitis) Mean ± SD	Group B (LC Only) Mean ± SD	Group C (Periodontitis Only) Mean ± SD	Group D (Healthy Controls) Mean ± SD	p-value
OHI-S	3.90 ± 0.38	3.50 ± 0.35	3.85 ± 0.36	0.65 ± 0.20	<0.001*
Russell's Periodontal Index	4.00 ± 0.43	0.40 ± 0.41	3.95 ± 0.40	0.30 ± 0.15	<0.001*
PPD (mm)	6.90 ± 0.65	2.40 ± 0.55	6.85 ± 0.60	0.00 ± 0.00	<0.001*
CAL (mm)	4.80 ± 0.75	0.00 ± 0.00	4.75 ± 0.70	0.00 ± 0.00	<0.001*

**Figure 3:**
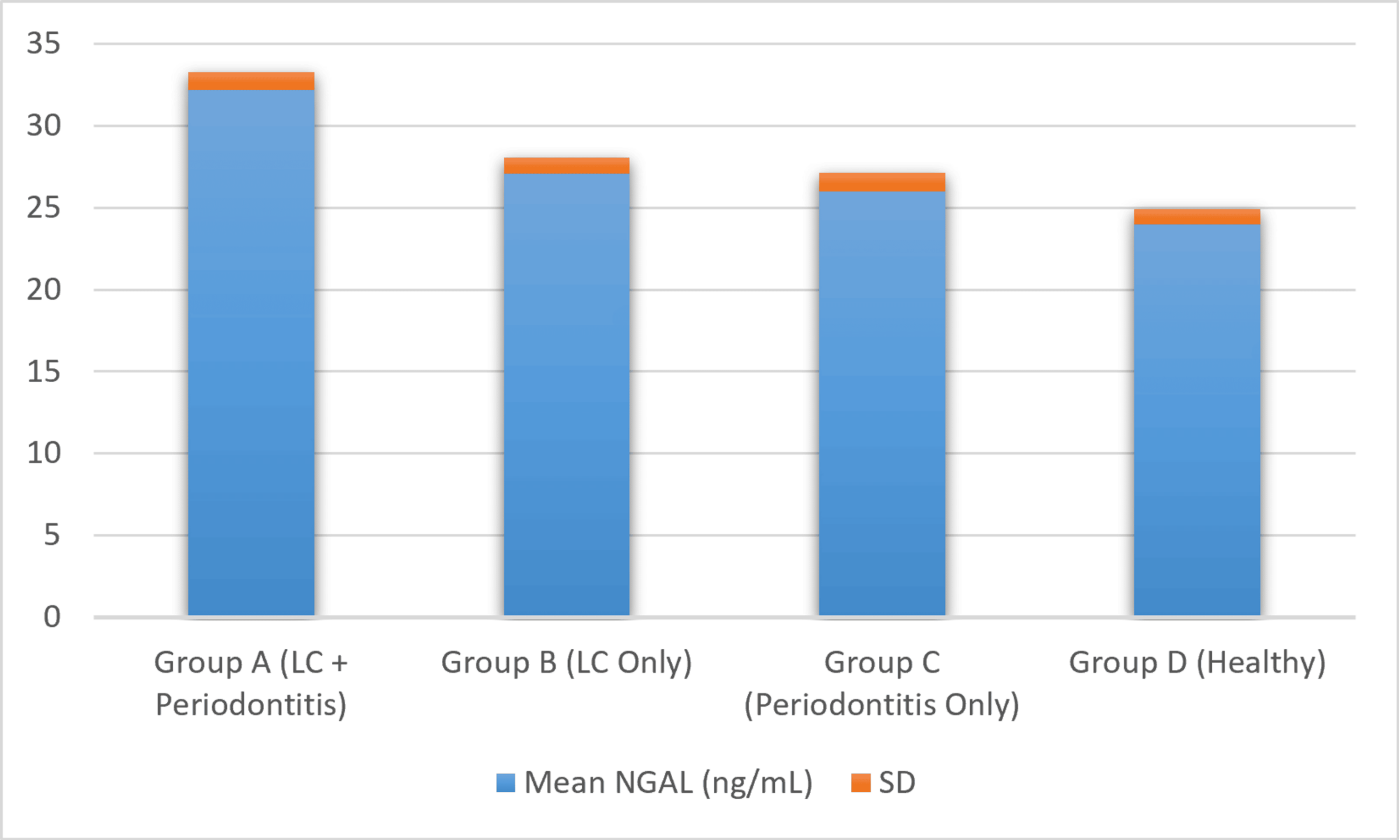
Mean salivary neutrophil gelatinase-associated lipocalin (NGAL) levels among the study groups

Table 4 and Figure 3 indicate that the comparison of mean salivary Neutrophil Gelatinase-Associated Lipocalin (NGAL) levels among the four study groups demonstrated a statistically significant difference. Group A exhibited the highest mean salivary NGAL level (32.2 ± 1.05 ng/mL), followed by Group B with a mean level of 27.1 ± 0.98 ng/mL and Group C with 26.0 ± 1.12 ng/mL. The lowest mean NGAL level was observed in Group D (24.0 ± 0.90 ng/mL). One-way ANOVA revealed a highly significant difference in salivary NGAL levels among the groups (F = 198.42, p = 0.0003), indicating a marked variation in NGAL expression across the study populations. In addition to statistical significance, effect size analysis demonstrated a very large effect of study group on salivary NGAL levels (η² = 0.943), indicating that approximately 94.3% of the variance in salivary NGAL concentrations was attributable to differences among the study groups.

**Table 4:** Comparison of mean salivary neutrophil gelatinase-associated lipocalin (NGAL) levels between various groups One-way ANOVA F = 198.42, p = 0.0003**

Groups	Mean (ng/mL)	SD	F value*	p-value
Group A- Liver Cirrhosis patients who have Periodontitis (n=10)	32.2	1.05	198.42	0.0003
Group B – Liver Cirrhosis Only (n=10)	27.1	0.98		
Group C – Periodontitis Only (n=10)	26.0	1.12		
Group D – Healthy (n=10)	24.0	0.90		

Post-hoc analysis revealed that Group A (liver cirrhosis with periodontitis) had significantly higher salivary NGAL levels than Groups B, C, and D (p < 0.01), indicating the greatest inflammatory burden when both conditions coexisted. Groups B (liver cirrhosis only) and C (periodontitis only) did not differ significantly from each other (p ≈ 0.08), suggesting that each condition independently contributes to elevated NGAL levels to a similar extent. However, both Groups B and C exhibited significantly higher NGAL levels than healthy controls (Group D). These findings support the association of salivary NGAL with both periodontal and hepatic inflammation, with the highest levels observed when the two inflammatory conditions are present simultaneously (Table 5).

**Table 5:** Post-hoc Tukey analysis for intergroup comparison *Post-hoc Tukey; statistically significant at p < 0.05

Study Group Comparison	Mean Difference (ng/mL)	p-value
A vs. B	5.1	<0.003
A vs. C	6.2	<0.002
A vs. D	8.2	<0.003
B vs. C	1.1	≈0.08
B vs. D	3.1	<0.003
C vs. D	2.0	<0.02

## Discussion

The present study evaluated salivary neutrophil gelatinase-associated lipocalin (NGAL) levels in patients with liver cirrhosis with and without periodontitis, periodontitis alone, and healthy controls and demonstrated a statistically significant difference among the groups. To the best of our knowledge, no previous study has evaluated salivary NGAL levels specifically in patients with concomitant liver cirrhosis and periodontitis, making this the first study to explore the combined impact of these two chronic inflammatory conditions on salivary NGAL expression. The highest NGAL levels observed in patients with concomitant liver cirrhosis and periodontitis suggest a cumulative inflammatory effect resulting from the coexistence of systemic and oral inflammatory burden. NGAL is a neutrophil-derived glycoprotein that is released in response to inflammation, infection, and tissue injury. Its role in periodontal disease has been previously investigated. Tan et al. reported significantly elevated NGAL levels in periodontal tissues and gingival crevicular fluid of patients with chronic periodontitis, suggesting its involvement in periodontal inflammation and connective tissue breakdown through regulation of matrix metalloproteinase-9 activity [12]. These findings support the increased salivary NGAL levels observed in the periodontitis-only group in the present study and confirm the relevance of NGAL as a marker of periodontal inflammatory activity. In liver disease, NGAL has been studied primarily as a marker of systemic inflammation and organ dysfunction. Mishra et al. demonstrated elevated NGAL levels in patients with liver cirrhosis and reported an association with disease severity and inflammatory status [13]. Similarly, Fagundes et al. observed increased NGAL expression in cirrhotic patients, particularly in the presence of bacterial infections and immune dysregulation [14]. These studies support the elevated salivary NGAL levels noted in the liver cirrhosis-only group in the present investigation, reflecting the chronic inflammatory and immunocompromised state associated with cirrhosis. However, none of the above studies evaluated NGAL levels in the context of coexisting periodontal disease and liver cirrhosis. The significantly higher NGAL levels in patients with both conditions in the present study indicate a possible synergistic effect, wherein periodontal inflammation may serve as a persistent peripheral source of inflammatory mediators, further amplifying systemic inflammation in cirrhotic patients. This observation strengthens the concept of an oral-systemic inflammatory link and highlights the importance of periodontal assessment in patients with chronic liver disease. Although NGAL has been widely studied in isolation in periodontal disease and liver cirrhosis, the lack of literature addressing their combined effect represents a critical research gap. The present study addresses this gap and suggests that salivary NGAL may serve as a potential non-invasive biomarker reflecting both periodontal and systemic inflammatory burden. The present study has certain limitations, including the small sample size and cross-sectional single-center design, which may limit the generalizability of the findings and prevent the establishment of causality. In addition, NGAL levels were assessed only in saliva at a single time point without correlation to disease severity, serum biomarkers, or microbiological and immunological parameters. Potential confounding factors such as smoking, alcohol consumption, medications, and oral hygiene practices were also not fully controlled. Despite these promising findings, larger longitudinal studies with age- and sex-matched cohorts are required to confirm the observed associations. Future investigations should also explore the relationship between salivary NGAL levels and the severity of both periodontal disease and liver cirrhosis to better define its potential clinical utility as a non-invasive biomarker in patients affected by these coexisting inflammatory conditions.

## Conclusions

In conclusion, the present study demonstrated significantly elevated salivary NGAL levels in patients with concomitant liver cirrhosis and periodontitis compared with other study groups, suggesting an increased combined inflammatory burden. These findings support the existence of an oral-systemic inflammatory link and highlight the potential of salivary NGAL as a non-invasive biomarker for assessing both periodontal and systemic inflammation.
